# Development and Optimization of a Cost-Effective Electrochemical Immunosensor for Rapid COVID-19 Diagnosis

**DOI:** 10.3390/bios15020067

**Published:** 2025-01-22

**Authors:** Thaís Machado Lima, Daiane Martins Leal, Zirlane Coelho Ferreira, Fernando de Jesus Souza, Danilo Bretas de Oliveira, Etel Rocha-Vieira, Helen Rodrigues Martins, Arnaldo César Pereira, Lucas Franco Ferreira

**Affiliations:** 1 Institute of Science and Technology, Federal University of the Jequitinhonha and Mucuri Valleys (UFVJM), Diamantina 39100-000, Minas Gerais, Brazil; thais.lima@ufvjm.edu.br (T.M.L.); daiane.leal@ufvjm.edu.br (D.M.L.); zirlane.coelho@ufvjm.edu.br (Z.C.F.); 2 Faculty of Medicine, Federal University of the Jequitinhonha and Mucuri Valleys (UFVJM), Diamantina 39100-000, Minas Gerais, Brazil; fernando.jesus@ufvjm.edu.br (F.d.J.S.); danilo.bretas@ufvjm.edu.br (D.B.d.O.); etel.vieira@ufvjm.edu.br (E.R.-V.); 3 Pharmacy Department, Federal University of the Jequitinhonha and Mucuri Valleys (UFVJM), Diamantina 39100-000, Minas Gerais, Brazil; helen.martins@ufvjm.edu.br; 4 Department of Natural Sciences, Federal University of São João del-Rei (UFSJ), São João del-Rei 36307-352, Minas Gerais, Brazil; arnaldo@ufsj.edu.br

**Keywords:** pencil graphite electrodes, electropolymerization, 4-hydroxybenzoic acid, SARS-CoV-2, electrochemical platforms

## Abstract

The coronavirus disease (COVID-19) pandemic has created an urgent need for rapid, accurate, and cost-effective diagnostic tools. In this study, an economical electrochemical immunosensor for the rapid diagnosis of COVID-19 was developed and optimized based on charge transfer resistance (Rct) values obtained by electrochemical impedance spectroscopy (EIS) from the interaction between antibodies (anti-SARS-CoV-2) immobilized as a bioreceptor and the virus (SARS-CoV-2). The sensor uses modified pencil graphite electrodes (PGE) coated with poly(4-hydroxybenzoic acid), anti-SARS-CoV-2, and silver nanoparticles. The immobilization of anti-SARS-CoV-2 antibodies was optimized at a concentration of 1:250 for 30 min, followed by blocking the surface with 0.01% bovine serum albumin for 10 min. The optimal conditions for virus detection in clinical samples were a 1:10 dilution with a response time of 20 min. The immunosensor responded linearly in the range of 0.2–2.5 × 10^6^ particles/μL. From the relationship between the obtained signal and the concentration of the analyzed sample, the limit of detection (LOD) and limit of quantification (LOQ) obtained were 1.21 *×* 10^6^ and 4.04 *×* 10^6^ particles/μL, respectively. The device did not cross-react with other viruses, including Influenza A and B, HIV, and Vaccinia virus. The relative standard deviation (RSD) of the six immunosensors prepared using the shared-pool sample was 3.87. Decreases of 22.3% and 12.4% were observed in the response values of the ten immunosensors stored at 25 °C and 4.0 °C, respectively. The sensor provides timely and accurate results with high sensitivity and specificity, offering a cost-effective alternative to the existing diagnostic methods.

## 1. Introduction

The coronavirus disease 2019 (COVID-19) pandemic has put public health systems worldwide under pressure due to shortages of test kits and supplies required to meet the growing demand from patients. Consequently, mass testing has become a crucial tool to combat the spread of the virus, thereby reducing the number of cases and fatalities [1,2,3]. The gold standard for diagnosing COVID-19 is detection of the SARS-CoV-2 genome using real-time polymerase chain reaction (qPCR) in clinical samples. However, the widespread implementation of this method is limited by extended analysis time, high cost, and the need for advanced equipment, specific reagents, and trained professionals [4,5,6].

In response to the urgent need for efficient, cost-effective, and rapid diagnostic tools, researchers have explored alternative approaches to complement or supplement the existing diagnostic methods. In particular, electrochemical sensors have emerged as a promising avenue for clinical diagnosis, owing to their ability to provide real-time results, minimal reagent/sample requirements, and cost-effectiveness. These attributes render them suitable for mass testing without requiring specialized expertise or sophisticated instrumentation [7,8,9,10,11,12,13]. A comparison of these electrochemical sensors with traditional methods revealed their potential advantages. Although qPCR is highly accurate, it is time-consuming, expensive, and requires specialized equipment and personnel. In contrast, rapid antigen tests, which offer quicker results, often compromise sensitivity and specificity, leading to the possibility of false negatives [14,15,16,17,18,19].

Electrochemical immunosensors provide timely results with high sensitivity and specificity. This is achieved by immobilizing specific antibodies on a sensitive transducer to monitor antigen–antibody interactions, allowing accurate recognition of the target virus, SARS-CoV-2, for accurate diagnosis of the disease. Optimizing the conditions of immobilization of the bioreceptor, as well as those of development and modification of the transducer, can improve analytical parameters such as sensitivity, selectivity, cost, and speed of detection. By detailing these aspects, we highlighted the potential impact of the proposed immunosensor as a valuable addition to the diagnostic tools available for COVID-19 testing.

Chemically modified electrodes allow control and monitoring of the physical and chemical properties of the surface, enabling the development of highly specific and selective transducers for simple and effective sensors and biosensors [20,21,22,23]. These devices generally consist of an electrode with a layer of material that modifies their properties. These materials can be conductive polymers, inorganic materials, or composites [24,25,26,27]. Conductive polymers (CP) have attracted significant scientific and technological interest because of their adaptability as intelligent materials capable of responding to specific stimuli. This adaptability allows them to function as sensors and actuators in various technological domains [28,29].

Electropolymerization, a technique that fosters the creation of polymeric films on electrode surfaces, has exhibited substantial potential for producing materials with distinctive electrical and electrochemical properties. This technique offers an alternative for the development of highly sensitive and selective electrochemical sensors [30,31]. Conductive polymers possess functional groups that make them excellent materials for the immobilization of biomolecules and facilitation of rapid electron transfer, rendering them ideal for biosensor fabrication [32,33,34]. Utilizing these polymers as functionalized platforms for biosensor construction and development can ensure superior sensitivity, selectivity, reproducibility, stability, and applicability compared with conventional unmodified electrodes [35]. The use of metallic nanoparticles in electrochemical sensors for electrode modification has also attracted the attention of researchers [20,36,37,38,39]. Silver nanoparticles (AgNPs) possess good conductivity, chemical stability, and catalytic activity [40,41,42,43,44,45,46]. Moreover, AgNPs are considered potent signal transducers because of their optical properties that enable easy binding to biomolecules [47].

In this context, this study proposes the development of an electrochemical immunosensor for diagnosing COVID-19 using pencil graphite electrodes (PGE) modified with polymer films from 4-hydroxybenzoic acid (4-HBA), which is referred to as PGE/poly(4-HBA). To the best of our knowledge, no previous study has explored the use of poly(4-HBA) for this purpose. Additionally, these platforms can be sensitized with AgNPs and specific antibodies to detect SARS-CoV-2 particles and ensure accurate and rapid disease detection. This immunosensor combines cutting-edge technologies in electrochemistry with nanomaterials to develop a reliable and accurate platform for diagnosing COVID-19.

## 2. Materials and Methods

### 2.1. Chemicals

The following chemicals were used in this study: sulfuric acid (98.08%) from Química Moderna and sodium hydroxide (99%), potassium phosphate monobasic (99%), and potassium phosphate dibasic (99%) from Dinâmica. Additionally, 4-hydroxybenzoic acid (98%), potassium hexacyanoferrate (II) trihydrate (≥99.5%), potassium ferricyanide (III) (≥99%), potassium chloride (≥99.0%), sodium borohydride, silver nitrate, bovine serum albumin (BSA), and sodium citrate were purchased from Sigma-Aldrich (São Paulo, Brazil).

The viral samples were provided by Prof. Dr. Leonardo Camilo de Oliveira from the Laboratory of Biosafety Level 3 (NB3) at ICB/UFMG. Ultraviolet radiation was used for inactivation. A suspension of Vero cells (mock) served as the negative control for the virus.

### 2.2. Electrochemical Measurements

The experiments were conducted in a single-compartment electrochemical cell with a volume of 1.0 mL. The working electrode, positioned at the bottom of the cell, was the PGE (0.0064 cm^2^). Ag/AgCl (KCl 3.0 M) was used as the reference electrode and a graphite wire served as the auxiliary electrode. All solutions were prepared using ultrapure water with a resistivity of 18.2 MΩ cm and a conductivity of 0.054 μS cm^−1^, obtained from the Master System MS 2000 (Gehaka, São Paulo, Brazil). Electrochemical measurements were performed using an Autolab potentiostat (PGSTAT128N model) (Metrohm AG, Herisau, Switzerland) equipped with an FRA32M module and connected to a computer running Nova 2.1.7.

### 2.3. Preparation, Conditioning, and Modification of PGE

Pencil graphite from Pentel (Diadema, Brazil), Hi-Polymer, HB type, with a diameter of 0.9 mm, was divided into two sections, and only the base area was coated with Risqué nail polish to define the working area. After drying for 30 min, the electrodes were polished using 600-grit sandpaper (3M, Sumaré, Brazil), washed, and sonicated in an ultrasonic bath for 5 min. Subsequently, the samples were dried under ultrapure N_2_ gas and connected to a conductive base support for measurements.

Before electropolymerization, the PGE underwent electrochemical pretreatment in a 0.10 M H_2_SO_4_ solution with a potential of −1.10 V applied for 100 s. Electropolymerization was performed using cyclic voltammetry (CV) with a 2.50 mM concentration of the monomer (4-HBA) in a 0.50 M H_2_SO_4_ solution. Initially, 25 consecutive potential cycles were applied at a scan rate of 50 mV/s within the potential range of 0.0–+1.4 V.

### 2.4. Electrochemical Properties of the PGE/poly(4-HBA)

The modified electrode (PGE/poly(4-HBA)) was characterized using CV and electrochemical impedance spectroscopy (EIS). CV measurements were performed in the potential range from −0.20 to +0.60 V at a scan rate of 50 mV/s in a 0.50 M H_2_SO_4_ and in a potassium ferro/ferricyanide solution of 5.0 mM containing 0.10 M KCl.

For EIS, the frequency range varied from 100 kHz to 10 mHz, with a sinusoidal excitation amplitude of 10 mV and application of open circuit potential (OCP). Nova 2.1.7 software was utilized to conduct the adjustment and fitting processes. For the unmodified PGE, an equivalent circuit represented by R_s_(Q_dl_[R_ct_W]) was employed, whereas R_s_(Q_dl_[R_tc_W])(R_ct2_Q_dl2_) was used for the modified PGE. In these circuits, R_s_ is the ohmic resistance of the solution, Q_dl_ is the electric double-layer capacitance, R_ct_ is the charge-transfer resistance, W is the Warburg impedance, R_ct2_ is the polymer resistance, and Q_dl2_ is the polymer capacitance.

To increase the sensitivity of SARS-CoV-2 detection on the electrochemical platform, we optimized the electropolymerization conditions for 4-HBA, considering the effects of monomer concentration, the number of potential cycles, and the scan rate. The optimized parameters were obtained using solutions with 2.5 mM monomer concentrations, 25 potential cycles, and a scan rate of 50 mV/s.

### 2.5. Synthesis and Charectarization of Silver Nanoparticles

The synthesis of silver nanoparticles (AgNPs) followed the methodology adapted from Jana et al. (2001) [48]. Briefly, a solution containing 45 mL of a 0.3 mM AgNO_3_ solution and 2.7 mM sodium citrate was prepared and stirred for one hour in an ice bath to maintain the temperature below 4 °C. Subsequently, 10 mL of 2.9 mM sodium borohydride (NaBH_4_) was added to the solution and kept in an ice bath for an additional 30 min. The color change from transparent to yellow indicated the formation of AgNPs. The solution was continuously stirred for 2 h in an ice bath, and the nanoparticles were washed and centrifuged at 4800 rpm for 2 h to remove excess salts and ions. After the synthesis, the AgNPs were stored in an amber bottle in a refrigerated environment. The concentration of the AgNPs was estimated as 0.25 mM.

The AgNPs were characterized by UV-Vis spectroscopy using a Shimadzu UV-2550 spectrometer with the AgNP solution diluted to 1:4. Parameters such as zeta potential, polydispersity index, and hydrodynamic diameter were determined in triplicate and without prior dilution using Zetasizer Nano ZS equipment (Malvern Instruments, Worcestershire, UK) at a temperature of 25 °C and a fixed angle of incidence of 173°. Dynamic light scattering (DLS) was used to determine the polydispersity index and hydrodynamic diameter. DLS, which is associated with electrophoretic mobility, was used to determine the zeta potential.

### 2.6. Fabrication of the Immunosensor

To immobilize AgNPs and SARS-CoV-2 antibodies (IgG) on PGE/poly(4-HBA), a solution of 1.0 µL of nanoparticles diluted in phosphate buffer (1:10) was added to 249 µL of the antibody solution diluted in phosphate buffer (1:250). Immobilization was achieved using the physical adsorption method, in which 15 µL of the nanoparticle-antibody solution was applied to the electrode surface for 30 min. Subsequently, the electrodes were washed with stirred phosphate-buffer solution and dried thoroughly. Next, 15 µL of 0.01% bovine serum albumin (BSA) solution was added to each electrode and incubated for 10 min. The electrodes were washed again in stirred phosphate-buffer solution. The immunosensor was then ready to react with positive and negative viral controls.

**Figure 1:**
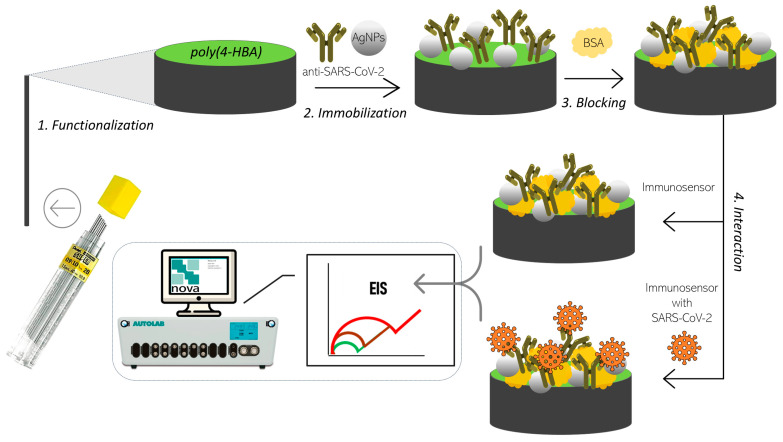
Schematic representation of the immunosensor preparation process. The steps included surface modification of the PGE with a polymer film derived from 4-hydroxybenzoic acid, immobilization of anti-SARS-CoV-2 antibodies with AgNPs, and subsequent blocking of the surface with BSA. The immunosensor was ready for the detection of SARS-CoV-2.

For the reaction, 15 µL of each positive or negative control solution, diluted at a ratio of 1:10 with phosphate buffer, was added to the immunosensor surface and incubated for 20 min. Following incubation, the electrodes were washed with phosphate buffer to remove unbound molecules. The preparation scheme of the immunosensor is shown in Figure 1.

Parameters such as antigen dilution and immobilization time have been previously examined to determine the optimal conditions for biomolecule immobilization via physical adsorption. Antibodies were tested at dilutions of 1:10, 1:50, 1:100, 1:250, and 1:500. After selecting the optimal concentration, immobilization was assessed at intervals of 5, 10, 30, 60, and 90 min. BSA was evaluated at dilutions of 1.0, 0.1, 0.01, and 0.001%. Subsequently, immobilization times of 5, 10, 15, 30, and 45 min were tested. Finally, the positive samples of SARS-CoV-2 were analyzed using the developed immunosensor at dilutions of 1:10, 1:50, 1:100, 1:250, and 1:500. After selecting the optimal dilution, analysis times were evaluated at 5, 15, 20, 60, and 90 min.

### 2.7. Determination of the LOQ and LOD of the Immunosensor

The calibration curve, constructed using different dilutions of a standard inactivated virus solution, served as the basis for establishing critical parameters such as the limit of detection (LOD) and limit of quantification (LOQ). The LOD was determined using Equation 3 × S_b_/*a*, while the LOQ was computed using the formula 10 × S_b_/*a*. In these expressions, S_b_ signifies the standard deviation of the blank solution and *a* represents the slope of the linear equation derived from the calibration curve at different virus concentrations.

### 2.8. Analysis of Clinical Samples and Cross Reactions

The immunosensor was tested using a pool of positive and negative samples of various dilutions (1:10, 1:20, 1:30, 1:40, 1:50, 1:100, and 1:500). Additionally, individual samples from 10 patients infected with SARS-CoV-2 and 10 non-infected patients were analyzed at a 1:10 dilution. The samples were subjected to qPCR analysis using the Center for Disease Control/USA protocol (catalog # 2019-nCoVEUA-01) [49].

This study was approved by the Ethics Committee (CEP) of the Federal University of the Jequitinhonha and Mucuri Valleys (UFVJM), protocol 4.557.181.

Moreover, the interference of other viruses, such as Influenza A and B, Vaccinia virus, and human immunodeficiency virus (HIV) was investigated at a dilution of 1:250 under optimal conditions. Alterations in electrochemical response were examined for each interfering virus.

## 3. Results and Discussion

### 3.1. Modification and Characterization of PGE

The PGE underwent electrochemical treatment prior to electropolymerization. Subsequently, electrochemical evaluation was performed using a solution of potassium ferro-ferricyanide in the same electrolyte. Notably, the entire PGE preparation process was performed manually, emphasizing the importance of performing electrochemical treatments on the electrodes. A thorough analysis of their electrochemical behavior is crucial to ensure the reproducibility of the results. Conducting studies in acidic media enhances the efficiency of assessing the potential interferents that may accumulate on the electrode’s surface. Applying a cathodic potential to the PGE minimizes the extensive oxidation of the material, thereby avoiding significant changes in the graphite’s original arrangement. As expected, PGE exhibited oxidation and reduction peaks in the K_4_Fe(CN)_6_/K_3_Fe(CN)_6_ profile of this redox couple. In the context of reversible processes, the expected difference in the peak potentials (∆E_p_) was 60 mV and the ratio of anodic to cathodic currents (*i*_pa_/*i*_pc_) was 1.0. Although the specific results are not shown, a ∆E_p_ value of 105 mV and *i*_pa_/*i*_pc_ of 0.94 were obtained. The deviation from the expected values can be attributed to the surface roughness of the graphite electrodes, which may hinder the transport of species during the process. Based on these analyses, it was concluded that the electrodes exhibiting this voltammetric profile would be suitable for future analyses. However, during the treatment step, some electrodes did not exhibit the desired profile, prompting them to be re-polished and subjected to a new pretreatment. If the discrepancy in the profile persisted, then the electrode was discarded.

**Figure 2:**
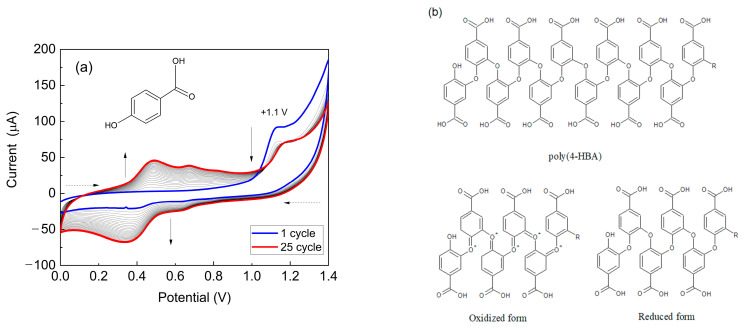
(**a**) Cyclic voltammograms obtained for PGE in 2.50 mM 4-HBA prepared in 0.50 M H_2_SO_4_. The CV was recorded over 25 potential cycles at a scan rate of 50 mV/s. The dashed arrows represent the potential sweep direction, whereas the solid arrows indicate the current direction as a function of the increasing number of potential cycles. Insert: 4-hydroxybenzoic acid. (**b**) Chemical structure proposed by Santos et al. (2019) for poly(4-HBA), including oxidized and reduced forms of the polymer. Adapted from [30]; permission conveyed through Copyright Clearance Center, Inc., Order number: 5923290001864.

Figure 2a illustrates the modification of PGE with poly(4-HBA) in acidic medium. In the first potential cycle, an irreversible oxidation peak at +1.1 V for 4-HBA was observed, corresponding to the monomer and formation of radical cations. Additionally, two redox couples appeared between +0.2 to +0.8 V, indicating the formation and deposition of an electroactive material on the PGE’s surface.

The decrease in the current from the second cycle onwards is likely attributed to the consumption of monomer species at the electrode/solution interface. As the number of potential sweeps increased, the current values of the redox peaks associated with the polymeric material also increased, suggesting that a greater amount of material was deposited on the electrode. These findings align with those of previous studies by our research group [30,33,50,51], where electropolymerization and the characterization of the polymeric films of this monomer were investigated using conventional graphite electrodes. The chemical structure of poly(4-HBA) proposed by Santos et al. (2019) is shown in Figure 2b [30].

**Figure 3:**
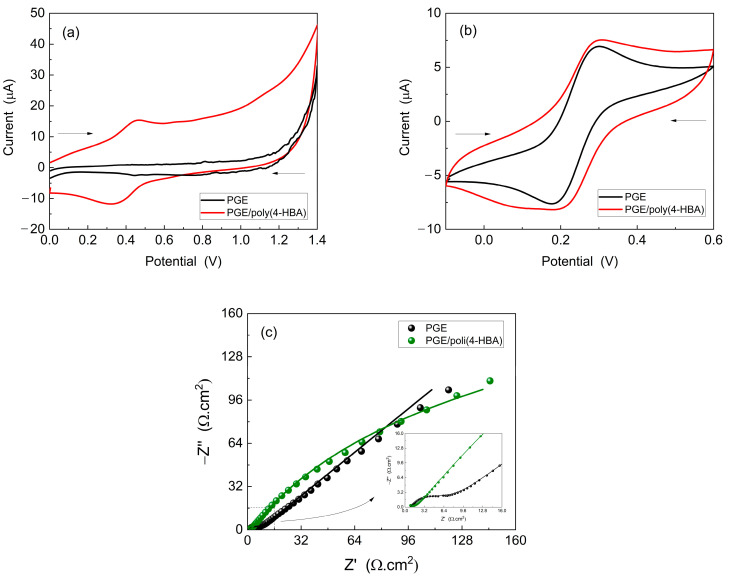
Cyclic voltammograms obtained for PGE and PGE/poly(4-HBA) at 50 mV/s in: (**a**) 0.50 M H_2_SO_4_; (**b**) 5.0 mM K_4_Fe(CN)_6_/K_3_Fe(CN)_6_ containing 0.10 M KCl; and (**c**) Nyquist diagrams of PGE and PGE/poly(4-HBA) in 5.0 mM K_4_Fe(CN)_6_/K_3_Fe(CN)_6_ containing 0.10 M KCl. E_ap_: OCP. Amplitude: 10 mV. Frequency: 100 kHz–10 mHz.

The electrochemical properties of PGE/poly(4-HBA) and unmodified PGE were investigated by immersing them in 0.50 M H_2_SO_4_ and potassium ferro-ferricyanide solutions. CV and EIS were employed for this analysis, and the results are shown in Figure 3.

In the case of CV analysis in 0.5 M H_2_SO_4_ (Figure 3a), the voltammogram of PGE/poly(4-HBA) exhibited increased electroactivity in the range from 0.2 to 0.6 V compared to that of the unmodified PGE, which showed only capacitive currents. A redox peak was observed in the same potential region as that observed during electropolymerization (Figure 2a), indicating the adsorption of an electroactive material related to poly(4-HBA). This electroactivity is related to the oxidized and reduced forms of poly(4-HBA), as shown in Figure 2b.

Figure 3b shows the CVs curves obtained for the potassium ferrocyanide/ferricyanide redox probe. Here, it was observed that PGE/poly(4-HBA) exhibited altered electrochemical behavior compared with unmodified PGE. The voltammogram of the modified electrode exhibits distinct peaks and current values, suggesting the influence of the polymeric film on the redox reactions of the electroactive species.

Figure 3c shows the Nyquist diagrams obtained for the potassium ferro-ferricyanide solution. An increase in the resistance to charge transfer (Rct) was observed for PGE/poly(4-HBA), as evidenced by the larger diameter of the semicircle in the high-frequency regions of the Nyquist plot. The semicircle represents the total current related to the Faradaic process and reflects the charge transfer between the electrode and the oxidized/reduced species in solution, corroborating the voltammetric measurements shown in Figure 3b. Additionally, both electrodes exhibited Warburg impedance (W or Zw), characterized by a straight line with a phase angle of approximately 45° in low-frequency regions. The presence of the Warburg impedance indicates a relative resistance to mass transfer between the diffusional layer and the oxidized/reduced species, thus limiting the current passage in the Faradaic pathway.

The results of this study show that PGE is a viable option for use as an electrochemical transducer in biosensors. The similar response observed between the PGE and conventional electrodes [30,33,50,51] suggests that the PGE is a reliable alternative that offers simplicity, versatility, affordability, and practicality as an electrochemical transducer.

### 3.2. Characterization of the AgNPs

The formation of AgNPs comprises three distinct phases: reduction, nucleation, and growth phases. In the reduction phase, the silver ions present in the solution are converted into metallic silver (Ag^0^), initiating the formation of the nanoparticle nuclei. In the nucleation phase, nuclei begin to collide with each other in a process that requires high energy and determines the size of the resulting nanoparticles. The reducing agent, NaBH_4_, acts as an effective disperse-protective agent, generating small nuclei. However, owing to the intensity of the reaction, the nuclei tend to aggregate, resulting in an increase in the size of the nanoparticles. To prevent AgNP agglomeration, a stabilizing agent such as sodium citrate (Na_3_C_6_H_5_O_7_) was introduced. This agent surrounds the nanoparticles, forming a self-organized layer that prevents agglomeration and guarantees the stability of the AgNPs [52].

The stabilizing agent can bind to the nanoparticles through two mechanisms: electrostatic interactions and steric hindrance. In the electrostatic interaction mechanism, the charged molecules of the stabilizing agent are distributed around the nanoparticles, forming stable structures that prevent the aggregation of other nanoparticles. In the case of steric hindrance, longer chains of the stabilizing agent interact with the surface of the nanoparticle, preventing other nanoparticles from approaching and bonding to this structure. In this proposed setup, the mechanism is attributed to electrostatic interaction, due to possible interactions between the free carboxyl sites present in the citrate structure, which can establish new electrostatic interactions, preventing the nanoparticles from aggregating [53]

The resonance of electrons on the surface of nanoparticles can be observed through an optical property called a Localized Surface Plasmon (LSP). Resonance is observed as an intense absorption of light in a certain range of wavelengths in the visible spectrum. When the nanoparticles were dispersed, LSP resonance was maintained, generating a characteristic color. When nanoparticles aggregate, their PSL resonance changes, generating a change in light absorption and causing a change in the color of the solution. SLP can be characterized using ultraviolet-visible (UV-Vis) absorption spectroscopy, which allows the absorption of light at different wavelengths. Analysis of the UV-Vis absorption spectrum provides information on the position of the PSL absorption band and its intensity, making it possible to determine the size and concentration of the nanoparticles [54].

**Figure 4:**
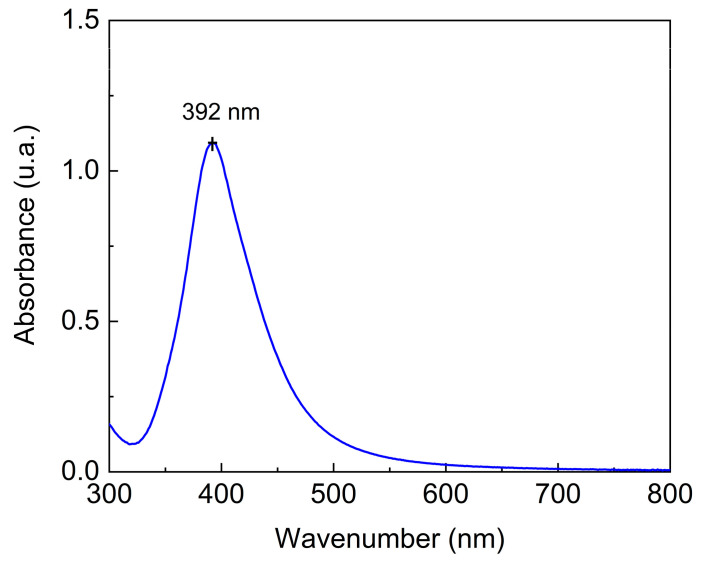
UV-vis absorption of AgNPs.

In Figure 4, the UV-Vis spectrum of AgNPs shows an absorption maximum at 392 nm, indicating the presence of AgNPs with particle diameters estimated at 50–70 nm [55]. DLS analysis was used to determine the possible aggregation of AgNPs and their zeta potential. The hydrodynamic diameter of the particle was 22.3 (±0.70) nm and the polydispersity index (PDI) was 0.57 (±0.01). The zeta potential was −7.3 (±0.92) mV. AgNPs consist of small particles ranging in size from to 1–100 nm [55]. According to the results obtained, the synthesized particles were sufficiently small to be characterized as AgNPs. The polydispersity index, which describes the degree of “non-uniformity” of the particles, was determined considering a range of 0.0 to 1.0, values that represent a perfectly uniform sample and a highly polydisperse one with multiple particle sizes, respectively. The PDI obtained was less than 0.57 when there was evidence of a broad particle size distribution profile. In contrast, the PDI observed was higher than 0.3, indicating highly dispersed formulations. Thus, a PDI of 0.57 can be considered as medium polydispersity [55].

The AgNPs showed a zeta potential close to neutral (between −10 and +10 mV). High zeta potential values, whether negative or positive, indicate a greater tendency for repulsion between the particles, which indicates good physical stability. The closer to neutrality, the greater the tendency for attraction between the nanoparticles, generating agglomeration, aggregation, and precipitation of the material, which leads to the instability of the nanosystem in dispersion. However, the average size of the AgNPs was relatively small, indicating that large aggregates were not formed.

### 3.3. Optimization of Transducer Modification Conditions

**Figure 5:**
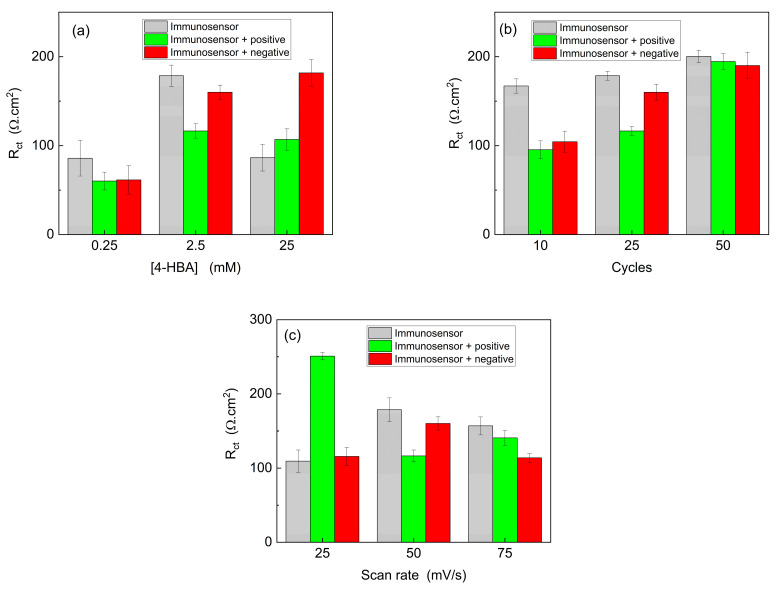
R_ct_ values as a function of: (**a**) monomer concentration; (**b**) potential cycles; and (**c**) scan rate used in the 4-HBA electropolymerization over PGE obtained for immunosensor; immunosensor + positive control, and immunosensor + negative control in 5.0 mM K_4_Fe(CN)_6_/K_3_Fe(CN)_6_ containing 0.10 M KCl. E_ap_: OCP. Amplitude: 10 mV. Frequency:100 kHz–10 mHz.

The monomer concentration plays a crucial role in the modification of pencil graphite electrodes because it directly affects mass transport and the electrogeneration of the polymeric film. Diffusion and adsorption processes facilitate the entry of monomer molecules into the pre-surface layer, where they are subsequently adsorbed by the electrode surface. During this process, the monomer is consumed, necessitating the influx of new molecules for the continuous formation of the polymeric film. Considering this, the effect of monomer concentration was investigated using concentrations of 0.25, 2.5, and 25 mM. Figure 5a illustrates that a monomer concentration of 0.25 mM yielded identical values for the positive and negative controls, although it exhibited a significant difference compared to the immunosensor. In contrast, the 25 mM dilution displayed distinguishable values between the immunosensor and the positive control, but the negative control exhibited much higher values. Therefore, in accordance with the expected pattern, a monomer concentration of 2.5 mM demonstrated a greater difference in the positive control than in the immunosensor, with the negative control signal closely resembling that of the immunosensor. Thus, the results obtained for this dilution suggested the capability of the device to recognize the target analyte.

In the presence of positive targets, there was a decrease in resistance to charge transfer. Typically, the opposite behavior is expected, where an increase in Rct values can be attributed to the interaction of immobilized antibodies with SARS-CoV-2 present in the sample on the surface of the immunosensor, thereby increasing the Rct values. However, we hypothesized that, because the immobilization of antibodies on the electrochemical platform is achieved through adsorption, upon contact with the virus, there might be a stronger interaction between the antibodies and the virus, leading to their removal from the immunosensor surface, resulting in lower Rct values.

Following the optimization of the monomer concentration, the number of potential cycles during electropolymerization was evaluated. This analysis is important because it is directly correlated with the amount of material formed on the electrode surface. It is assumed that a higher number of cycles will lead to the generation of a greater number of polymeric films. The electrodes were modified using 10, 25, and 50 potential cycles in ferrocyanide/potassium ferricyanide solution. As shown in Figure 5b, after 10 cycles, similar values were obtained for both positive and negative controls, which were distinct from the immunosensor signal. After 50 cycles, excessively high resistance values were obtained, which made it impossible to differentiate the samples. Therefore, upon analyzing the modified electrodes after 25 cycles, it was determined that the amount of film formed was adequate for distinguishing the positive from the negative control. Consequently, 25 potential cycles were performed for the PGE modification using 2.50 mM 4-HBA prepared in 0.50 M H_2_SO_4_.

The scan rate employed during the electrochemical polymerization was further optimized. Electropolymerization involves the formation of monolayers or the arrangement of polymeric films on the electrode surface. Hence, scanning speed is a crucial parameter for analysis, because different speeds are associated with various types of polymeric film organization on the electrode surface [30,52]. Figure 5c shows the influence of the scan rate on the PGE modification. Using 25 and 50 mV/s, a notable difference was observed between the positive and negative controls, with similar values obtained for the immunosensor and negative control. At 75 mV/s, a high value was observed for the negative control, possibly indicating the disorganization of the film. The most favorable response was obtained at 50 mV/s, enabling differentiation between the positive and negative samples in a shorter time compared to the equivalent at 25 mV/s. Consequently, after optimizing the parameters for electrode modification, the optimal conditions were determined to be a monomer concentration of 2.5 mM, 25 potential cycles, and a scan rate of 50 mV/s.

### 3.4. Optimization of the Immunosensor Response

**Figure 6:**
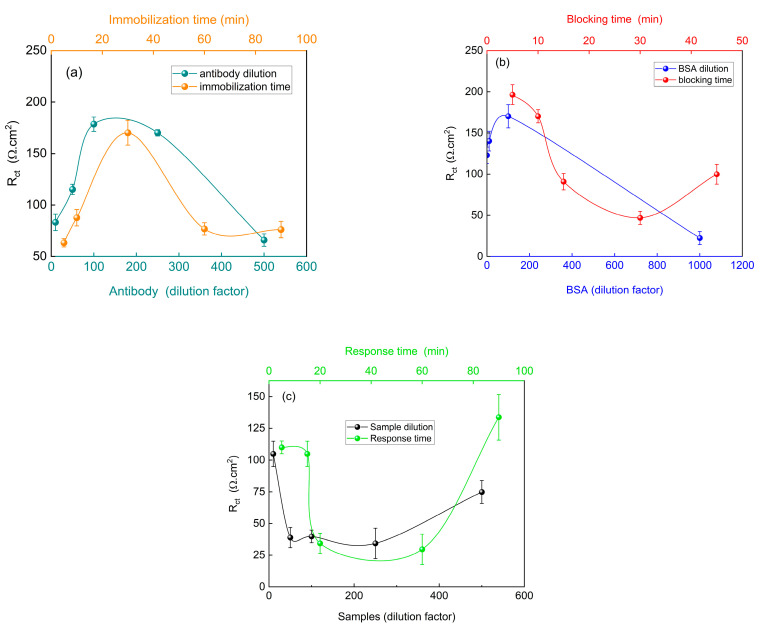
R_ct_ values as a function of (**a**) antibody dilution and immobilization time, (**b**) BSA dilution and blocking time, and (**c**) sample dilution and response time of the immunosensor. EIS measurements were obtained in 5.0 mM K_4_Fe(CN)_6_/K_3_Fe(CN)_6_ containing 0.10 M KCl. E_ap_: OCP. Amplitude: 10 mV. Frequency:100 kHz–10 mHz.

Following the evaluation of the effectiveness of the proposed immunosensor, the steps involved in the construction and response of the device were optimized, specifically focusing on the immobilization of the bioreceptor, surface blocking with BSA, and the response to the samples used, as shown in Figure 6.

Initially, the concentration of the anti-SARS-CoV-2 antibodies immobilized on the modified PGE was optimized. Figure 6a demonstrates an increase in charge transfer resistance between the 1:10 and 1:100 dilutions, with relative stability observed at the 1:250 dilution. However, a decrease in R_ct_ was observed at a dilution of 500. When assessing immobilization time, the highest R_ct_ value was obtained after 30 min. As shown in Figure 6b, both the 10 min immobilization time and 100-fold dilution exhibited the highest R_ct_ values. After interaction of the virus present in the samples with the immunosensor (Figure 6c), a decrease in R_ct_ was observed. The lowest resistance to charge transfer was observed at a 1:250 dilution, resulting in a greater separation between the virus signal (positive control) and the immunosensor signal. This trend was also observed for the immobilization times of 20 and 60 min. Greater differentiation between the immunosensor signal and the immunosensor with the virus reduces the risk of false positive diagnoses.

Although indications suggest that the proposed immunosensor can detect SARS-CoV-2 at various stages of the disease, corresponding to different viral load levels, the most efficient diagnosis was achieved at a 1:250 dilution. Furthermore, while the 60 min immobilization time yielded the best result, the difference compared to the 20 min time was minimal. Considering that this study investigated the use of the proposed device as a rapid test for SARS-CoV-2, conducting a diagnosis within 60 min is impractical, whereas obtaining reliable results within 20 min is feasible.

### 3.5. Determination of LOD and LOQ of Imunossensor

**Figure 7:**
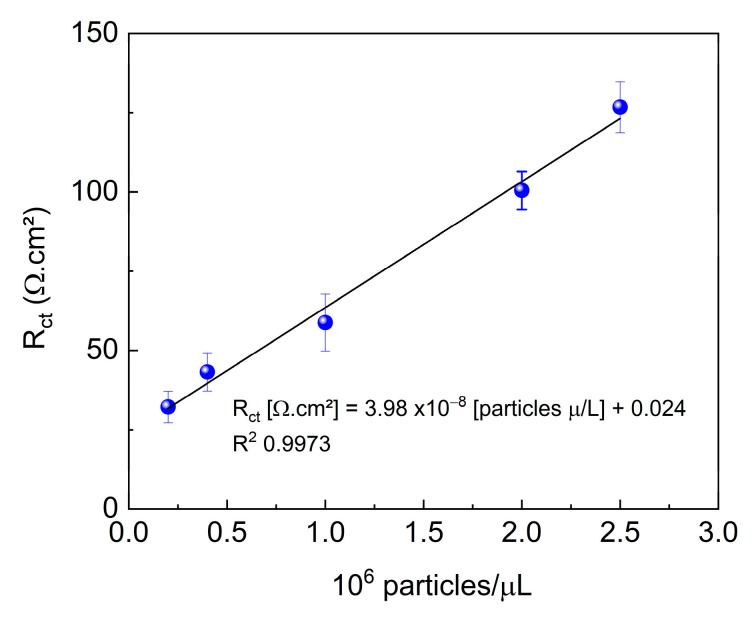
R_ct_ values as a function of immunosensor + positive samples at different concentrations. EIS measurements were obtained in 5.0 mM K_4_Fe(CN)_6_/K_3_Fe(CN)_6_ containing 0.10 M KCl. E_ap_: OCP. Amplitude: 10 mV. Frequency:100 kHz–10 mHz.

Figure 7 shows the response of the immunosensor to different dilutions of the standard sample. As the concentration of the evaluated sample increased, detection became more sensitive. The increase in Rct with increasing quantity of viruses is directly related to blocking of the electrode surface. This phenomenon occurs because, upon binding to the immobilized antibodies, the viruses form a virus-antibody complex layer that acts as a physical and electrical barrier, hindering electron transfer between the electrode and the electrolyte. This layer partially blocks the active sites of the electrode, reducing the available area for redox reactions and, consequently, increasing resistance. Furthermore, because both viruses and antibodies are poorly conductive materials, the blocking effect is intensified, contributing to an increase in Rct.

The immunosensor responded linearly in the range of 0.2 to 2.5 × 10^6^ particles/μL. The relationship between the obtained signal and the concentration of the analyzed sample was used to determine the LOD and LOQ. The resulting values were 1.21 × 10^6^ and 4.04 × 10^6^ particles/μL for LOD and LOQ, respectively.

Compared with other impedimetric biosensors, Wang et al. [56] developed an LSG-based device that achieved a linear detection range for SP-RBD from 150 pM to 15 nM with a sensitivity of 0.0866 [log(M)]^−1^ and a limit of detection (LOD) of 7.68 pM. Southe et al. [57] described the use of a label-free electrochemical biosensor based on the covalently conjugated SARS-CoV-2 spike protein for POC detection of COVID-19 antibodies, obtaining an LOD of 21 ng/mL. An LOD of 150 ng/mL was achieved by an impedimetric immunosensor developed using the immobilization of antibodies on reduced graphene oxide (rGO) [58]. An immunosensor based on gold nanostructured carbon electrodes (AuNS/SPCEs) for detecting the SARS-CoV-2 nucleocapsid protein (N-protein) in saliva showed a linear range of only 0.1−100 ng/mL. The calculated LOD was 174 pg/mL [59].

The vast majority of studies available in the literature focus on the detection of the SARS-CoV-2 Spike (S) protein. The present study aimed to detect viral particles, making it difficult to compare the obtained LOD with those reported in the literature. Thus, the determination and quantification of SARS-CoV-2 viral particles using an impedimetric immunosensor was a distinguishing feature of this study.

### 3.6. Application of the Immunosensor in Clinical Analysis

**Figure 8:**
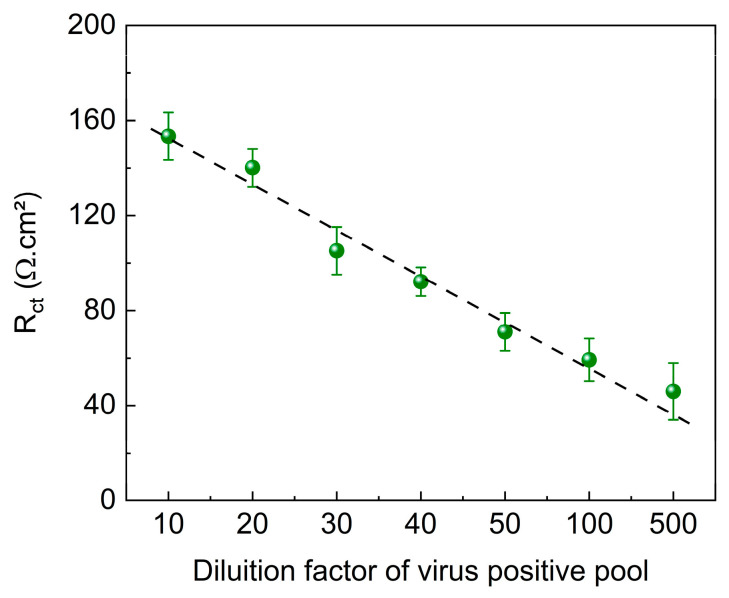
R_ct_ values as functions of immunosensor + positive pools. EIS measurements were obtained in 5.0 mM K_4_Fe(CN)_6_/K_3_Fe(CN)_6_ containing 0.10 M KCl. E_ap_: OCP. Amplitude: 10 mV. Frequency: 100 kHz–10 mHz.

The immunosensor was evaluated using clinical samples obtained from patients infected with SARS-CoV-2. To assess the potential effects of the sample matrix, pools containing positive and negative samples were analyzed at various dilutions (1:10, 1:20, 1:30. 1:40, 1:50, 1:100, and 1:500). The results of these analyses are shown in Figure 8.

Figure 8 shows a linear trend for the different dilutions of the positive pool samples, with a decrease in the R_ct_ value as the dilution factor increased. Despite the presence of other substances in human nasal samples that could potentially interfere with the system, the matrix effect did not interfere with virus identification and quantification.

**Figure 9:**
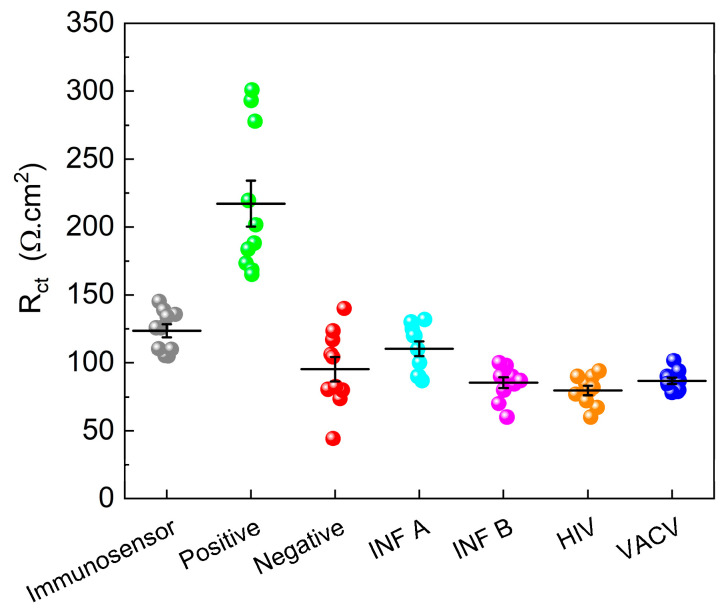
R_ct_ values as functions of immunosensor and immunosensor with positive pool, negative pool, Influenza A (INF A); Influenza B (INF B); human immunodeficiency virus (HIV), and Vaccinia virus (VACV). EIS was performed in a 5.0 mM K_4_Fe(CN)_6_/K_3_Fe(CN)_6_ containing 0.10 M KCl. E_ap_: OCP. Amplitude: 10 mV. Frequency: 100 kHz–10 mHz.

A total of 20 samples, consisting of ten positive samples and ten negative samples validated by qPCR, were analyzed. In addition, the potential interference of other viruses, such as HIV, IFA, B, and Vaccinia, was investigated. The results, shown in Figure 9, revealed that the R_ct_ values of the negative samples closely resembled those of the immunosensor, suggesting minimal interaction between the negative samples and immunosensor surface. Conversely, the positive samples demonstrated an increase in the Rct value, indicating the successful detection of SARS-CoV-2. Notably, interfering viruses decreased the R_ct_ value, underscoring the specificity of the immunosensor for SARS-CoV-2 detection.

To establish a reliable diagnostic threshold, an Rct value of approximately 160 Ω.cm^2^ was identified. Samples with Rct values exceeding this threshold were classified as positive, whereas those with R_ct_ values below this threshold were considered negative. This threshold-based approach offers a straightforward and effective means of distinguishing between positive and negative samples, ensuring accurate diagnosis of SARS-CoV-2 infection.

The decision to analyze individual clinical samples at a 1:10 dilution was based on the improved differentiation between positive and negative samples at this dilution, providing a more robust and reliable diagnosis of SARS-CoV-2 infection.

Notably, the developed immunosensor exhibited excellent selectivity, as it did not show significant sensitivity to interfering viruses, such as HIV, IF A, INF B, and VACV. This selectivity is of utmost importance to ensure an accurate diagnosis and prevent false-positive results. By carefully optimizing the concentrations and immobilization times of biomolecules and implementing a threshold-based approach, the immunosensor achieved reliable diagnostic performance with a clear distinction between positive and negative samples. The established threshold value of approximately 160 Ω.cm^2^ provides a practical and objective criterion for interpreting results and facilitating the implementation of immunosensors in clinical settings.

Immunosensor reproducibility refers to the ability to obtain consistent and reproducible results when the tests are performed using the same device. On the other hand, repeatability is a measure of the consistency of results when the same test is performed several times with the same sample, by the same operator, and under the same conditions. Considering the data in Figure 9, a relative standard deviation (RSD) of 3.87 was obtained for six different immunosensors manufactured using the same pool sample, demonstrating the good performance of the constructed device. Operational stability is related to the ability of a device to maintain its functional properties over time and under specific storage and usage conditions. In this study, we investigated the drop in immunosensor response when stored at room temperature (±25 °C) and at 4 °C for 45 days. Decreases in the response values of 22.3% and 12.4% were obtained for the ten immunosensors stored at 25 °C and 40 °C, respectively. This suggests a good storage capacity; however, for application purposes, it is a parameter that needs to be optimized and improved.

Overall, this study contributes to the field of diagnostic biosensors for infectious diseases, particularly for SARS-CoV-2 detection. The developed electrochemical immunosensor demonstrated favorable characteristics including ease of construction, low cost, rapid analysis time, and robust performance in clinical analysis. The findings presented here pave the way for further advancements in the field of biosensing technologies, offering valuable insights into the development of reliable and efficient diagnostic tools for the timely identification and management of infectious diseases. The developed platforms can be incorporated into mobile devices for quick virus detection tests in healthcare settings, such as hospitals, clinics, and pharmacies. This integration enables the immediate identification of viruses, even in areas with limited resources, without the need for specialized equipment or trained personnel.

## 4. Conclusions

This study aimed to develop an electrochemical immunosensor capable of detecting SARS-CoV-2. The pencil graphite electrodes were modified with conductive polymers derived from 4-hydroxybenzoic acid to serve as transducers for immobilizing anti-SARS-CoV-2 antibodies and AgNPs. The conditions for the electropolymerization of the polymeric film were determined to be a 2.50 mM monomer concentration in 0.50 M sulfuric acid as the support electrolyte, with 25 potential cycles at a scan rate of 50 mV/s. The concentration and immobilization times of the biomolecules were optimized. Anti-SARS-CoV-2 antibodies were immobilized at a concentration of 1:250 for an immobilization time of 30 min, and the optimal concentration for blocking BSA was determined to be 0.01% for 10 min. The immunosensor demonstrated better detection of the virus at a 1:10 dilution within a 20 min timeframe and showed no sensitivity to interfering viruses, such as Influenza A and B, HIV, and Vaccinia.

The immunosensor was subjected to different dilutions of the standard sample and showed a linear response in the range of 0.2 to 2.5 × 10^6^ particles/μL, with values of 1.21 × 10^6^ and 4.04 × 10^6^ particles/μL for the LOD and LOQ, respectively.

Although the presence of other substances in human nasal samples could potentially interfere with the system, the matrix effect did not compromise virus identification and quantification. The immunosensor exhibited a linear trend for different dilutions of the positive pool samples, with a decrease in the Rct values as the dilution factor increased. In the analysis of individual samples and possible cross-reactions, other viruses did not interfere with the immunosensor response, highlighting their specificity for detecting SARS-CoV-2.

The developed immunosensor offers numerous advantages such as straightforward assembly, practicality, cost-effectiveness, and swift analysis. Its high sensitivity and specificity make it a valuable tool for the detection of SARS-CoV-2 in clinical samples. This heightened sensitivity and selectivity resulted from a thorough optimization of the functionalization parameters of the proposed transducer, coupled with fine-tuning of bioreceptor immobilization.

## Data Availability

Data supporting the results of this study are included in this article.
